# Dystroglycan and Mitochondrial Ribosomal Protein L34 Regulate Differentiation in the *Drosophila* Eye

**DOI:** 10.1371/journal.pone.0010488

**Published:** 2010-05-05

**Authors:** Yougen Zhan, Nadia Y. Melian, Mario Pantoja, Nicola Haines, Hannele Ruohola-Baker, Charles W. Bourque, Yong Rao, Salvatore Carbonetto

**Affiliations:** 1 Centre for Research in Neuroscience, McGill University Health Centre, Montreal, Quebec, Canada; 2 Department of Biochemistry and Institute for Stem Cell and Regenerative Medicine, University of Washington, Seattle, Washington, United States of America; Medical College of Georgia, United States of America

## Abstract

Mutations that diminish the function of the extracellular matrix receptor Dystroglycan (DG) result in muscular dystrophies, with associated neuronal migration defects in the brain and mental retardation e.g. Muscle Eye Brain Disease. To gain insight into the function of DG in the nervous system we initiated a study to examine its contribution to development of the eye of *Drosophila melanogaster*. Immuno-histochemistry showed that DG is concentrated on the apical surface of photoreceptors (R) cells during specification of cell-fate in the third instar larva and is maintained at this location through early pupal stages. In point mutations that are null for DG we see abortive R cell elongation during differentiation that first appears in the pupa and results in stunted R cells in the adult. Overexpression of DG in R cells results in a small but significant increase in their size. R cell differentiation defects appear at the same stage in a deficiency line *Df(2R)Dg^248^* that affects *Dg* and the neighboring mitochondrial ribosomal gene, *mRpL34*. In the adult, these flies have severely disrupted R cells as well as defects in the lens and ommatidia. Expression of an *mRpL34* transgene rescues much of this phenotype. We conclude that DG does not affect neuronal commitment but functions R cell autonomously to regulate neuronal elongation during differentiation in the pupa. We discuss these findings in view of recent work implicating DG as a regulator of cell metabolism and its genetic interaction with *mRpL34*, a member of a class of mitochondrial genes essential for normal metabolic function.

## Introduction

Dystroglycan (DG) was first identified in brain as a receptor for laminin [1], [2] and subsequently cloned from skeletal muscle as a component of the dystrophin glycoprotein complex (DGC) that links the membrane cytoskeleton to the extracellular matrix [3]. Mice with DG-deficient skeletal muscle develop a progressive muscular dystrophy consistent with the notion that DG forms the functional core of the DGC [4], [5]. Selective deletion of the DG gene in brain leads to defects in morphogenesis, including malformation of cortical lamina and neuronal migration errors similar to mutations that affect glycosylation and ligand binding of α-DG [6], [7]. Several congenital muscular dystophies, referred to as dystroglycanopathies [8], [9] are associated with reduced ligand binding of DG and are characterized by defective neuronal migration (lissencephaly), severe mental retardation and eye anomalies [10], [11], [12], [13]. The brain defects are thought to result from aberrant attachment to and assembly of the basement membrane at the radial glial-ependymal interface [6], [7]. However, glial driven basement membrane aberrations may not be sufficient to explain the neural defects [14] seen in DG-null brains. Indeed, in type 1 lissencephaly there is increased cell death and impaired neural differentiation [15]. Moreover, in Large^myd^ mice, with hypoglycosylated and dysfunctional α-DG, the hindbrain has disorganized and missing nuclei [16]. Since DG is expressed in radial glia and neurons, it is also unclear whether neurons and glia contribute to the brain phenotypes.

Many proteins serving fundamental cellular functions are well retained with evolution, as for example, eyeless and its mammal ortholog Pax-6, which are required for eye formation from flies to mammals [17]. Moreover, in *Drosophila* the main components of the DGC are conserved [18]. *Drosophila* DG has been shown to be involved in progressive muscular dystrophy [19], changes in larval muscle attachment and sarcomere size [20], as well as in formation of the posterior cross vein of the wing [21]. Here we show that DG is necessary for neuronal differentiation in the fly eye where it functions R cell autonomously and appears independent of supporting cells responsible for extracellular matrix deposition [22]. mRpL34 is a protein involved in ribosomal protein translation and encoded by a class of genes responsible for mitochondrial diseases that typically lead to muscle and brain disorders [23]. Interestingly, mutations in the *Drosophila mRpL34* gene exacerbate the R cell phenotype in double *Dg, mRpL34* mutants. Moreover recent evidence has revealed a novel function for DG in regulating cell metabolism during oocyte development [24]. Similar regulation in neurons could have major implications for DG function in neural development and even behavior [25].

## Results

### Dystroglycan is expressed on the apical surface of R cells following neuronal commitment

In vertebrates there is a single DG transcript for a protein that interacts with its major ligands via O-linked carbohydrates that decorate its mucin-like domain [26]. Mab IIH6, which recognizes functional carbohydrate side chains in vertebrates, will label *Drosophila* DG expressed in heterologous cells (Text S1, Fig. S1). Moreover, mutations in genes that O-glycosylate vertebrate and *Drosophila* DG lead to muscle disruption [8], [20], [27], [28] suggesting significant conservation of structure and function of the mucin-like domain, though the glycosylation pathways are not identical.

**Figure 1:**
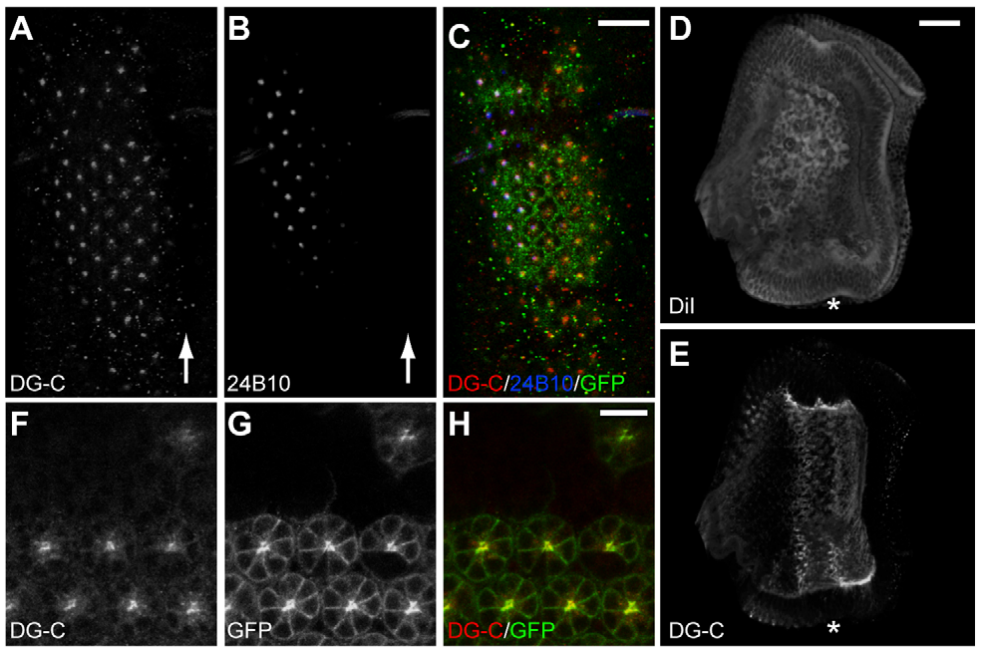
Dg is expressed by R cells during eye development. Third instar larval (A–E) and pupal (F–H) eye discs stained with DG-C antibody. A–C, third instar eye disc with a small *Df(2R)Dg^248^* clone, identified by lack of GFP (C, green), stained with anti-DG-C (A, red in C) and 24B10 (B, blue in C). Areas lacking GFP also lack DG-C staining demonstrating specificity of the antibody (A and C). Posterior to the morphogenetic furrow (left of arrow) DG-C is concentrated at the apical surface of R cells that are identified by 24B10 expression (B and C, blue). (D–E) whole mount wild type third instar disc stained with DiI to highlight membranes (D) and DG-C (E), viewed onto the basal surface of the disc. DG-C (E) localizes to the basal surface of the disc, in the anterior region, and at the morphogenetic furrow. (F–H) eye disc at 40% pupal development carrying a small *Df(2R)Dg^248^*clone, identified by lack of GFP (G and H, green). GFP positive wild type R cells, highlighted by the membrane bound GFP (G and H, green), localize DG-C (F and H, red) to the apical surface. In A–C the scale bar is 20 µm, D–E the scale bar is 25 µm and the arrows or asterisks indicates the position of the morphogenetic furrow with the posterior region to the left and anterior region to the right. Scale bar in F–H is 10 µm.

*Drosophila Dg* is subject to differential splicing of its mRNA to generate three isoforms [29]. With an antibody that is specific to the DG-C isoform that contains the entire mucin like domain [29] we mapped the distribution of DG-C in 3^rd^ instar larval and early pupa eye discs. To confirm the specificity of the antibody we generated eye discs mosaic for a deletion *Df(2R)Dg^248^* using FLP/FRT mitotic recombination driven by the *eyeless* (*ey*) promoter [30]. *Df(2R)Dg^248^* is a small deletion that removes the upstream regulatory region of *Dg*, resulting in loss of *Dg* function as well as part of a neighboring gene, *mRpL34* [24], [31]. In wild type neurons in mosaic eye discs DG-C antibody staining was localized to the apical surface but was absent from the *Dg* mutant cells (Fig. 1A, C; F–H), confirming that the antibody selectively recognizes DG. When discs were co-stained with 24B10, a marker for R cells, newly committed R cells can be seen just posterior to the morphogenetic furrow (Fig. 1A, B, arrows). DG staining was observed before cells became positive for the neuronal marker 24B10, indicating that DG expression precedes commitment to a neural cell fate. In addition to its localization on R cells DG was also present at the basal surface of the eye discs in the anterior region prior to and at the morphogenetic furrow (Fig. 1E). In early pupa (40% pupal development) DG staining was found at the apical surface of neurons with less intense labeling at the lateral surfaces of R cells (Fig. 1F–H). We saw a similar pattern in discs and pupal eyes stained with an antibody that recognizes all the DG isoforms (Fig. S2).

### DG is necessary for neural differentiation

**Figure 2:**
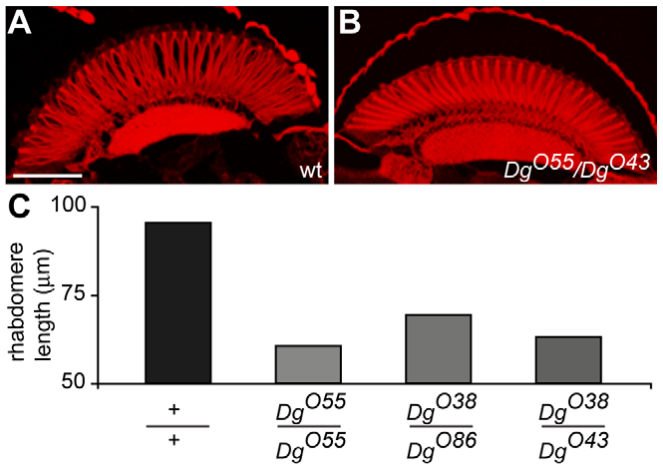
Dg mutants have thin retinas. (A, B) Transverse retinal sections of adult eyes stained with F-actin to highlight the R cell rhabdomeres. The retina of *Dg^O55^/Dg^O43^* (B) is thinner than wild type (A) and R cells are obviously shorter. (C) Chart shows mean rhabdomere lengths in wild type and *Dg* mutants (wild type n = 30; *Dg^O55^/Dg^O55^* n = 51; *Dg^O38^/Dg^O86^* n = 23; *Dg^O38^/Dg^O43^* n = 14). Scale bars in (A) represents 75 µm.

**Figure 3:**
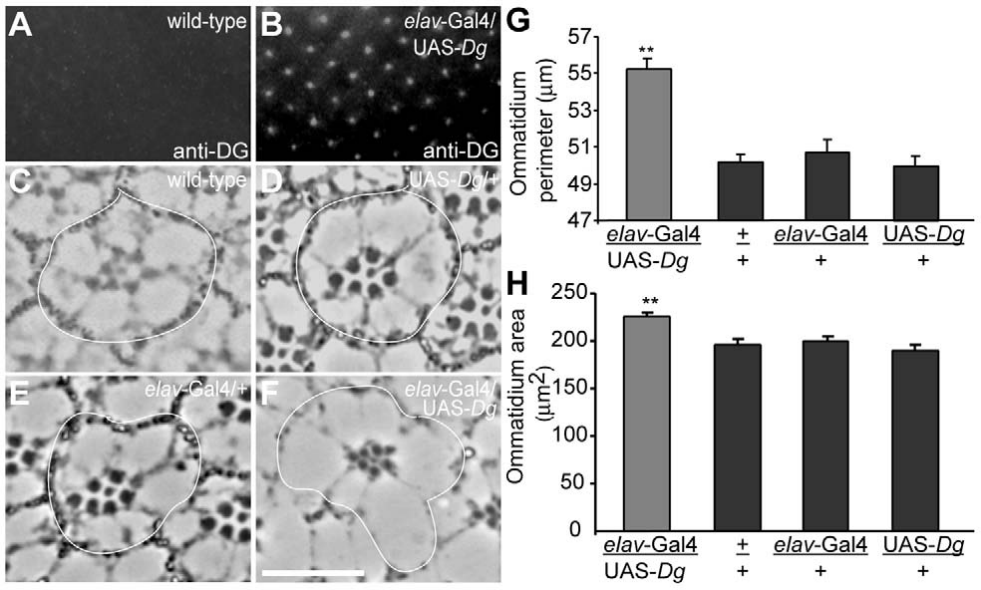
Over-expression of Dg-C in R cells increases ommatidial size. Over-expression of *Dg*-C was demonstrated with a concentration of anti-DGcyto antibody too low to detect wild type levels of DG (A) in third instar discs that revealed clear labeling of omatidia in transgenic flies (B). Cross sections of ommatidia (outlined) in wild type flies (C) and flies containing UAS- *Dg*-C/+ alone (D), *elav*-Gal4/+ alone (E) and *elav*-Gal4/UAS-*Dg*-C (F) showed that over-expression of *Dg*-C in R cells induces bigger ommatidia and expansion of R cells (F) as compared with the controls (C–E). The number of R cells remains unchanged (F). Quantification of these data shows that the ommatidia perimeters (G) are slightly but significantly increased when *Dg*-C is over-expressed in R cells driven by *elav*-Gal4 as are ommatidial areas (H). **, P<0.01. Bar, 10 µm.

Several point mutations null for *Dg* [21] have recently been generated and the adult flies have obvious behavioral phenotypes with abnormal wing posture and chronic shaking. To assess whether there were defects in CNS development we examined the fly eye and found that R cells were shorter than normal (Fig. 2A, B). Quantification revealed significant difference in R cell length in all *Dg* EMS point mutants examined (Fig. 2C). Wild type retinas (Fig. 2A) stained for F-actin to highlight photoreceptor rhabodomeres have 94 µm thick retinas whereas *Dg* mutants have retinas that were approximately 1/3 thinner (Fig. 2B) with some variance among the mutant lines (Fig. 2C). Thus DG appears to regulate R cell elongation. In support of this conclusion R cells were slightly, but significantly, enlarged when *Dg*-C was over-expressed in R cells of wild type animals (Fig. 3). In this instance DG is being driven in neurons alone suggesting an R cell autonomous effect of DG in regulating size.

### Genetic interaction of *Dg* with *mRpL34*

**Figure 4:**
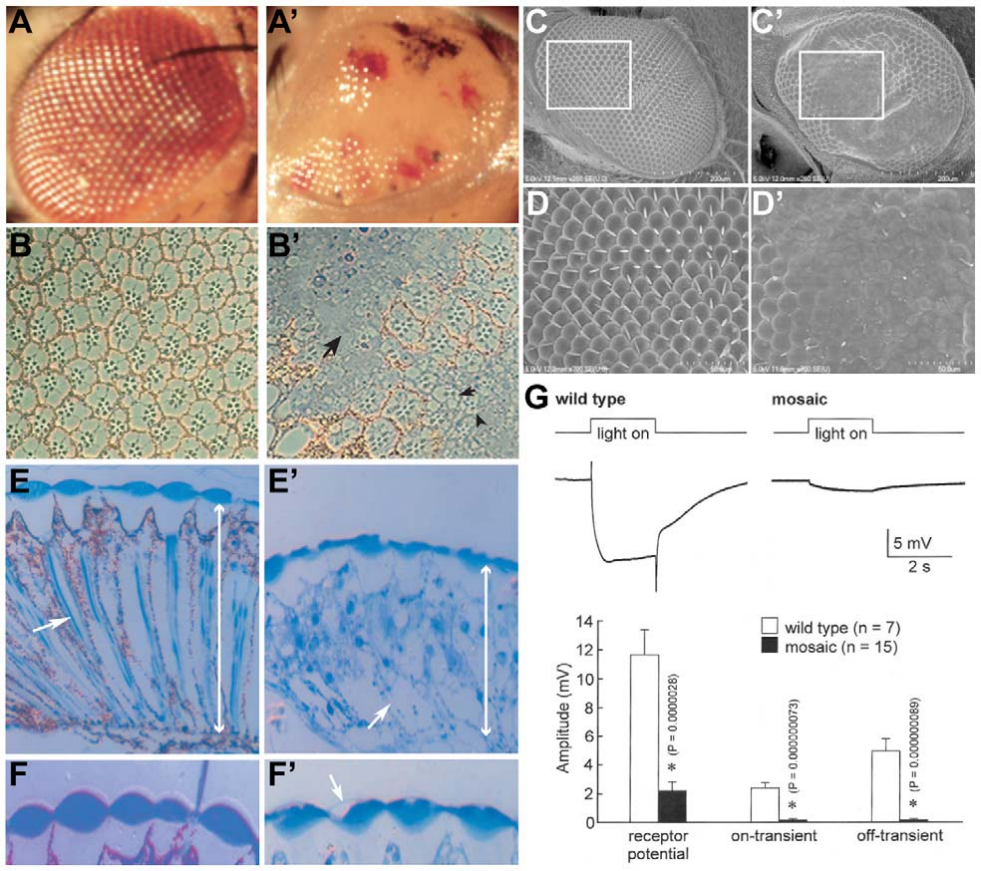
Large mosaic clones of *Df(2R)Dg^248^* results in disruption of the adult eye. Light and scanning EM micrographs of the wild type fly eye (A–F) show the classical ommatidial facets and bristles of the external eye. A′–F′, show the equivalent micrographs of fly eyes of *Df(2R)Dg^248^* clones generated by the *ey*, FLP/FRT system. The *Df(2R)Dg^248^* tissue in A′ is white while tissue with heterozygous expression of the wild type gene is red. The *Df(2R)Dg^248^* (white) regions appear flattened and glossy (A′). Cross sections of the internal eye reveal that the normal (B) array of R-cells with central rhabdomeres is disrupted (B′ arrows) and there appears to be cell debris (B′ arrowhead) in these regions. Electron micrographs reveal regions where the external eye has collapsed and the facets have been obliterated (C′, D′). A transverse section through the eyes reveals the normal length (E, double arrow head) of the retina and its ordered array of ommatidia (arrow). The *Df(2R)Dg^248^* (white) region appears disrupted (arrow) and the retinal thickness is shorter (double arrow head). The lenses, which normally (F) form a biconvex disc, are flattened on their external, but not internal, faces though the cuticle that forms the external boundary is visible within a flattened and partially “empty” lens (arrow, F′). (G) ERGs were recorded after a 5 min dark adaptation followed by a 2 sec bright-light pulses (top trace). The bottom trace shows representative ERGs of wild type (left) and *Df(2R)Dg^248^* regions (white) of mosaic eyes. In *Df(2R)Dg^248^* regions the 9-14 mV R cell depolarization (left) was greatly diminished and the early and late synaptic transients (left) completely abolished. Quantification of ERG parameters in wild type and *Df(2R)Dg^248^* patches showed significant differences (P<0.01) between wild type and *Df(2R)Dg^248^* eyes.

Mitochondrial dysfunction results in a subset of neurodegenerative diseases because of the function of this organelle in apoptosis. Other mitochondrial diseases have been linked to mutations in genes encoding respiratory chain proteins and, more recently, nuclear genes like *mRpL34*, that are responsible for mitochondrial ribosomal translation [23], [32]. Mitochondrial diseases typically affect brain and muscle [23] similar to dystroglycanopathies [11]. For mitochondrial diseases this is thought to reflect the high metabolic activity of muscle and brain. Recently, Mirouse et al., [24] have reported that DG regulates cell polarity during development via AMP Kinase, a central regulator of metabolic homeostasis in cells [33]. In this context, we asked whether a similar pathway might function in CNS development by comparing R cell differentiation in *Df(2R)Dg^248^* with *Dg* point mutants. Since the *Df(2R)Dg^248^* homozygotes die as immature larvae we examined mosaic eyes generated using *ey*-FLP/FRT system [30]. In the adult *Df(2R)Dg^248^* tissue can be recognized by a lack of pigment granules and appear as white patches (Fig. 4A′). Mosaic eyes are irregularly shaped and appear glossy (Fig. 4A′). Electron micrographs show regions where the external eye has collapsed and the facets have been obliterated (Fig. 4C′, D′). Histology of the eye reveals flattening of the external aspect of the lens (Fig. 4E′–F′), which is normally a biconvex disc (Fig. 4E–F). Areas containing *Df(2R)Dg^248^* ommatidia, marked by the absence of brown pigment granules, appear disorganized (black arrowhead, Fig. 4B′). Importantly, the R cells are disrupted in this deficiency to an extent greater than the *Dg* point mutants where the R cells were simply stunted.

Electroretinograms (ERGs) were recorded from white *Df(2R)Dg^248^* areas, and red wild type regions of mosaic eyes as well as wild type eyes (Fig. 4G). ERGs from *Df(2R)Dg^248^* regions had greatly reduced R cell currents and lacked on and off transients (Fig. 4G) indicative of disrupted photo transduction and synaptic transmission in the brain [34]. The residual response is likely due to current spread from wild type regions of these mosaic eyes.

**Figure 5:**
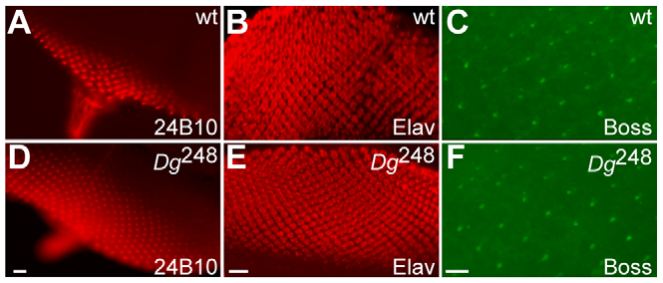
Third instar development is not disrupted in *Df(2R)Dg^248^* mosaics. Immunohistochemistry with antibodies to 24B10 (A, D), Elav (B, E) and Boss (Bride of Sevenless) (C, F) identify R cells in wild type (A–C) and *ey*/FLP induced mosaic eye imaginal discs (D–F) in the late 3rd instar larvae. 24B10 (A and D) and Elav (B and E), localize normally (A, B) in *Df(2R)Dg^248^* deficient eye discs (D, E). Similarly Boss, which is expressed on the apical surface of R8 and necessary for formation of R7, is localized normally (C) in *Df(2R)Dg^248^* deficient eye discs (F). Bar represents 10 µm.

To trace the genesis of these defects we examined 3^rd^ instar larval eye discs. R cells are sequentially specified and recruited to form presumptive ommatidia posterior to the morphogenic furrow. Wild type ommatidia are composed of the eight R cells, each roughly occupying an equal fraction of the omatidial volume, and attached to other R cells at their lateral and apical surfaces. During pupal development the R-cells elongate as they differentiate. Using neuronal and cell polarity markers R-cell specification and morphology were examined in *Df(2R)Dg^248^* mosaic eye discs. In third instar larvae, R cells were specified normally, as determined by 24B10 and Bride of Sevenless (Boss) staining of eye discs (Fig. 5D–F), suggesting that neither *Dg* nor *mRpL34* are required for neural commitment. Similar results were found with flies null for *Dg* (*Dg^O55^*; Melian and Carbonetto, unpublished). We conclude that lack of *Dg* alone or *Dg* plus *mRpL34* does not affect neuronal commitment that occurs in the 3^rd^ instar larva.

**Figure 6:**
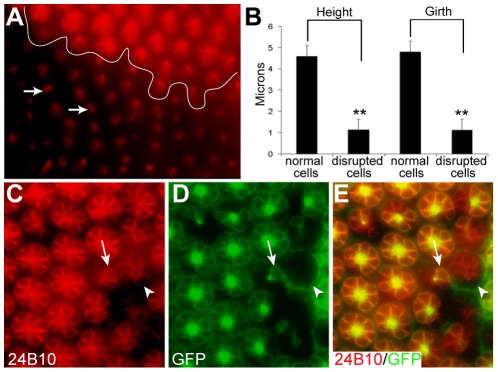
*Df(2R)Dg^248^* is required for photoreceptor organization, growth and survival during pupal development. A) An antibody, 24B10, was used to identify R cells in eye discs of 20% pupal development, mosaic for wild type and DG-deficient cells generated by FLP/FRT system. A line in (A) separates a disrupted region from a normal region. In 20 such preparations there were many small R cells (arrows, A) adjacent to patches with normal ommatidia. (B) Quantification of the lengths of lateral (apical to basal axis) membranes and (circumferential) basal membranes of R cell showed that those in disrupted regions were significantly shorter (**, P<0.01). C–E) 20% pupal development eye disc with small clones of *Df(2R)Dg^248^* cells. Individual R-cells labeled for 24B10 (C). *Df(2R)Dg^248^* clones are identified by a lack of GFP labeling (D). The merge (E) reveals the morphology of the *Df(2R)Dg^248^* photoreceptors. An ommatidium composed of only *Df(2R)Dg^248^* R cells (arrowhead) shows disorganized R cells. A single wild type R cell (arrow) has a normal morphology within an otherwise disorganized *Df(2R)Dg^248^* ommatidium.

We first observed developmental defects in *Df(2R)Dg^248^* flies during pupariation. Immunocytochemistry of mosaic discs at ∼20% of pupal development with 24B10 revealed aberrant R cell morphology (Fig. 6A, C–E). Instead of the typical pattern of eight R cells per ommatidium in wild type eyes (Fig. 6A, C–E) some *Df(2R)Dg^248^* deficient discs had ommatidia with few or no R cells (Fig. 6A). In experiments in which mosaic eye discs were generated with the *ey* FLP/FRT system, that favors production of small clones, *Df(2R)Dg^248^* deficient R cells are irregularly spaced within an ommatidium and are disorganized (Fig. 6C–E). In ommatidia composed of *Df(2R)Dg^248^* R cells 24B10 labeling revealed disorganized R cells (Fig. 6C, arrowhead) that had lost their polarity (Fig. 6C, S3). In an ommatidium composed of one wild type R cell surrounded by *Df(2R)Dg^248^* R cells (arrow, Fig. 6C), the wild type R cell's morphology is normal and appears indistinguishable from those in an ommatidia comprised entirely of wild type cells. It appears that expression of DG in a single R cell is sufficient to allow proper differentiation. Quantification of the lengths of lateral (apical to basal axis) membranes and (circumferential) basal membranes of *Df(2R)Dg^248^* mutant R cell clones (as in Fig. 6A) showed that average height of R cells at 20% pupal development is normally 4.59±0.52 µm and the basal surface is about 4.79±0.49 µm in diameter. In *Df(2R)Dg* ^248^ deficient discs both of these dimensions are reduced to 1.2±0.50 µm (Fig. 6B), suggesting that differentiation of *Df(2R)Dg* ^248^ R cells is abortive. This is consistent with the stunted R cells evident in *Dg* point mutation and taken together the overexpression data suggest that neural differentiation is R cell autonomous.

### Mutations in *mRpL34* are responsible for the external eye defects and neural degeneration in the adult

**Figure 7:**
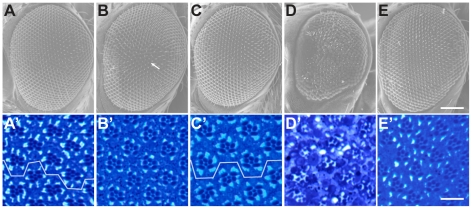
An mRpL34 transgene rescues external eye defects associated with *Df(2R)Dg^248^*. SEM of two day old female adult eyes (A–E) and the corresponding histology (A′–E′). A wild type eye shows the ordered array of ommatidia both on the surface (A) and within the retina (A′). On the surface, a *Dg^043^* eye (B) has a small region (arrow) of mispatterning of ommatidia. Within the *Dg^043^* retina (B′) the patterning of the retina is normal. The surface of a *Dg^086^* eye (C) as well as the within the retina (C′) shows a normal pattern of ommatidia. The *Dg^086^* eye shape is more spherical relative to wild type. A *Df(2R)Dg^248^* mosaic eye has a grossly disrupted array of ommatidia on its surface (D) as well as within (D′). Expression of an *mRpL34* transgene in a *Df(2R)Dg^248^* mosaic eye significantly rescues the external and internal eye disruption (E and E′). The scale bars are 100 µm (A–E); 10 µm (A′–E′). Line marks the retinal equator in A′ and C′.

To isolate the contribution of *mRpL34* to eye development we used flies expressing an *mRpL34* transgene in the *Df(2R)Dg* ^248^ background thus rendering the eye deficient solely in *Dg* [24]. Eyes from these flies were compared with *Dg^043^* (Fig. 7B, B′), and *Dg^086^* (Fig. 7C, C′) by scanning election microscopy (SEM). The SEM of *Df(2R)Dg^248^*+*mRpL34* flies revealed a normal array of ommatidia and normally shaped eye. Some subtle differences from the wild type were detected in the *Dg* point mutant lines, as for example *Dg^043^* eyes which had a slight mispatterning of the ommatidia (Fig. 7B arrow). SEM of *Dg^086^* (Fig. 7C) showed a normal pattern of ommatidia, but the overall eye was slightly misshapen and appeared smaller, relative to the wild type. The internal pattern of ommatidia is normal in these *Dg* deficient retinas (Fig. 7B′–C′) and the ommatidia maintain planar polarity (Fig. 7B′–C′). Consistent with the *Dg* point mutants, R cells had defects in polarity as revealed by disrupted targeting of β catenin to adherens junctions (Fig. S3). These data suggest that, while disruption of the R cell differentiation is clearly exacerbated by *mRpL34* and cell polarity is disrupted in the developing R cells, the flattened lens and omatidial alterations seen in *Df(2R)Dg^248^* (Fig. 2) is due to loss of *mRpL34* alone.

## Discussion

The fly genome has many of the components of the vertebrate dystroglycan complex [35] and reflecting this, flies with decreased levels of DG or dystrophin develop a progressive muscular dystrophy [19]. In mice lacking DG in the brain [7] or in humans with hypoglyosylated DG [8] there are profound defects in neuronal migration (lissencephaly, heterotopias). These diseases, collectively referred to as dystroglycanopathies, (Walker Warburg Syndrome, Muscle Eye Brain Disease, Fukuyama Congenital Muscular Dystrophy, Congenital Muscular Dystrophy Types 1C, 1D and Limb Girdle Muscular Dystrophy 2I) result in mental retardation and are often accompanied by eye defects [8]. Initial studies suggested that these lissencephalies result from a failure of radial astroglia endfeet to establish or stabilize the basement membrane at the pial surface [7] thus affecting migration of neurons that accumulate as heterotopias outside of the brain parenchyma. The interactions of DG in the nervous system are distinct from those in muscle [36] as a result we have focused on early events in neuronal development to understand those fundamental functions.

In flies DG is expressed during neural commitment in the 3^rd^ instar larva. However in both *Dg* point mutants in the deficiency line, *Df (2R) Dg248*, we were unable to detect any differences between mutants and wild-type eye discs in the expression of neuronal markers or in cell polarity. Thus it appears that DG is not necessary for neuronal commitment (Scherbata et al., 2007) although we did not see any defects in axonal projections (Fig. S2) to the brain (c.f. Scherbata et al., 2007). During pupal development the fly eye expands and thickens considerably. This involves elongation of R cells along with accessory cells. Mutations in *Dg* generate properly organized but thinner retinas with stunted R cells (Fig. 2). Our data indicate that these defects are R cell autonomous and appear initially during early pupal development. First, histological analysis of the adult eye indicates that R cells alone are affected. Second, expression of DG in R cells results in a small but significant increase in cell size (Fig. 3). Finally, in the *Df(2R)Dg^248^* line which is severely hypomorphic for *Dg* [24], [31] and null for *mRpL34* [24] R cells in the pupa do not elongate properly (Fig. 6B). Mosaic analysis during pupal development reveals null R cells are that disorganized (Fig. 6C–E arrows) within omatidia comprised of wild-type supporting cells. Conversely, we see examples of well-oriented, wild type R cells within ommatidia devoid of other photoreceptors (Fig. 6C–E). That these developmental defects are R cell autonomous in the double mutant *Df(2R)Dg^248^* line together with data noted above suggest strongly that it obtains for DG alone.

A corollary of the R-cell autonomous function of DG is that the defects in neurons likely do not result from defects in basement membrane assembly as in the vertebrate CNS [7]. Neurons in vertebrates as well as in flies do not form basement membranes hence their absence in the parenchyma of the vertebrate brain or neuropil of invertebrates lack basement membranes. Our preliminary data using MARCM analysis support this conclusion since we see no disruption in the basement membrane per se, which appears normal in 3^rd^ instar larvae as well as in the proximal region of the pupa in mosaic eyes (Melian and Carbonetto, unpublished)(Fig. S2, Fig. 6). At this point we favor the interpretation that the effects of DG are primarily intrinsic to neurons. Are there similar defects in neural differentiation in vertebrates apart from the disrupted migration and basement membrane assembly? Possibly, since lissencephalies have been reported to affect neural differentiation [37] and there is emerging evidence of defective neuronal differentiation in the retina and brains of mice with DG loss of function mutations [38].

How might the R cell autonomous function(s) of DG in neuronal differentiation occur? Poulton and Deng [39] have found that EGF receptor is in the DG pathway in signaling polarity in the fly oocyte and Shcherbata et al [19] reported that fly *Dg* interacts genetically with the insulin receptor in the eye. Yatsenko et al [40] have shown that a point mutation in a SH3 domain binding site abolishes the function of *Dg* in epithelial cell polarity. It will be interesting to determine whether this binding site in the cytoplasmic domain is critical for eye development. A DG lacking any extracellular sequence whose function is mediated by this SH3 binding site would argue strongly that DG is not simply a structural complex but initiates intracellular signaling.

Two recent papers [24], [31] have reported that DG contributes to loss of cell polarity in developing flies. Of special interest, Mirouse and coworkers (2009) have shown that DG is required under conditions of energetic stress. There are no mutations in *mRpL34*, a mitochondrial ribosomal protein, and these authors used a combination of double *Dg*/*mRpL34* mutants with transgenic expression of *mRpL34* or *Dg* to demonstrate co-dependence of cell polarity on both *Dg* and *mRpL34*. Since genes that encode mitochondrial proteins compromise oxidative phosphorylation [41], [42] Mirouse et al (2009) reasoned that this might be physiologically relevant. Interestingly, these mutations in mitochondrial genes typically result in a glossy eye phenotype as a result of diminished number of cone and accessory cells that are responsible for secreting the lens [42]. The numbers of R cells and their axonal projections to the brain is unaffected [42] as noted above for deficiency lines that affect *mRpL34* and *Dg* (Fig. 4, S2). Furthermore, defects in the external eye (lens, bristles) are attributable to lack of *mRpL34* as transgenic expression of this gene in the deficiency line rescues the lens and bristle organization (Fig 7E) though the R cells are somewhat disorganized (Fig. S3). Mutations in *Dg* alone cause abortive differentiation and stunted R cells (Fig. 2). In contrast the R cells in the deficiency lines are severely disrupted and seem to have degenerated (Fig. 6, 7D′) suggesting that *Dg* and *mRpL34* interact to regulate neuronal differentiation and survival. This not only implicates mitochondrial genes in post mitotic differentiation but is consistent with other data implicating these two genes in the same pathway of cell differentiation (Mirouse et al., 2009) and extends previous work in vitro that has implicated DG in cell survival [43], [44].

In keeping with a role for DG in regulation oxidative phosphorylation in mitochondria, Mirouse et al. (2009) show that embryos null for *Dg* and energetically stressed by glucose restriction or lacking both *Dg* and *mRpL34* develop follicle cells that are severely disorganized. The disorganization of follicular cells is strikingly similar to R cells lacking both *Dg* and *mRpL34* (Fig. 6). This novel pathway of DG regulation of cellular energy homeostasis involves AMPKinase [24] is restricted to stages of development much later than germline differentiation but may be common to many tissues including the nervous system. Neurons may be especially exploitative of this pathway. Takeuchi et al report altered behavior in a *Dg* hypomorph with a higher metabolic rate [25]. Curiously, these flies prefer lower ambient temperatures than wild-type flies. Thus the ambient metabolic state of the nervous system appears subject to control by DG and to impact behavior. It would be important to know whether similar ambient fluctuations in energy levels impact neural development and dystroglycanopathies

Many mitochondrial diseases affect both muscle and brain similar to mutations that alter DGC function [23]. From this point of view it is not surprising then that mutations in *mRpL34*, which is a nuclear gene encoding a ribosomal protein, exacerbated the *Dg* phenotype in R cells. That is R cells in the *Df(2R)Dg* ^248^ deficiency line are substantially more disrupted when compared with *Dg* point mutants. There is also a gross disruption of external eye development that is attributed solely to *mRpL34* since: A) it is rescued by expression of the *mRpL34* transgene in the *Df(2R)Dg* ^248^ line B) the *Dg* point mutants have only a very mild external eye defects (Fig. 7) C) Mutations in other genes that play role in oxidative phosphorylation in the mitochondria display a similar glossy eye phenotype [41], [42], [45]. Interestingly, some dystroglycanopathies are accompanied by eye defects (Martin, 2006) and one might speculate that this involves DG mediated energetic stress that is mimicked in the fly by dual mutations in *Dg* and *mRpL34*.

In several instances the fly eye has proven a valuable model of vertebrate development [46]. For DG, it appears that the conservation of function from fly to mammals is substantial [19]. Indeed, mice rendered null for DG by epiplast injection have grossly disrupted retinas similar to our studies with chimeric mice deficient in DG (Melian and Carbonetto, unpublished). It is, of course, difficult to determine how faithfully the cellular interactions in the vertebrate eye mirror those in the fly but there is good reason presented here and elsewhere that it will prove fruitful in uncovering hitherto novel mechanisms of DG function that will be valuable in exploring the severe muscular dystrophy, eye defects and mental retardation that accompanies dystroglycanopathies in humans.

## Materials and Methods

### *Drosophila* Strains

*Dg^248^* referred to here as *Df(2R)Dg^248^* is described [24], [31].The *w; FRT42D Dg^248^/Cy*O [31](Dr. H. Ruohola-Baker) is ready for clonal analysis as described below. The EMS *Dg* mutants *Dg^038^*/*Cy*O, *Dg^043^/Cy*O, *Dg* ^055^/*Cy*O, *Dg^086^*/*Cy*O [21] (Dr. R. Ray) were used directly as the stocks become homozygous. UAS-*Dg*-C as described [31](Dr. H. Ruohola-Baker). The *mRpL34* transgene *P[w^+^, mRpL34^+^]42.1/TM6B, Hu, Tb* [24](Dr. R. Ray) and *w; FRT42D Dg^248^*/*Cy*O were used to construct *w; FRT42D Dg^248^/Bc; P[w^+^, mRpL34^+^]52.1/Tb* to be used to generate clones as described below.

### Induction of *Dg*-deficient clones

Homozygous *Df(2R)Dg^248^* clones were generated by using ey-FLP/FRT [30] system. The *y w, GMR-LacZ ey-FLP; FRT42D LW^+^/Bc* was used to generate large clones and the *y w, GMR-LacZ ey-FLP; FRT42D GMR-myr-GFP* was used to generate small clones (Bloomington Stock Center). To generate eyes composed solely of *Df(2R)Dg^248^* the mosaic system *w*; *FRT42 GMR-Hid Cl2/Cy*O; *ey-gal4, UAS-FLP* [47] (Bloomington Stock Center) was modified to *y w, GMR-LacZ ey-FLP; FRT42D GMR-Hid Cl2/Cy*O. The mosaic systems were crossed with *FRT42D Dg^248^/Bc* or *w; FRT42D Dg^248^/Bc; P[w^+^, mRpL34^+^]52.1/Tb* as described above.

### Staining and Imaging

Immunofluorescence of eye discs was performed following standard procedures [48]. Primary antibodies were used as follows: rabbit anti-DG^ex8^ (1/50)[49], rabbit anti-Dg^cyto^ (1/1000)[31], mouse mab 24B10 1/250 Developmental Studies Hybridoma Bank [DSHB]), mouse anti-Boss (1/100 DSHB), rat anti-Elav (1/250, DSHB) mouse anti-β-catenin (Armadillo) (1/100, DSHB) and mouse anti-GFP (1/100 Invitrogen). Dapi was used to label nuclei. Tissue was also stained by DiI (1/200 Invitrogen) or Phalloidin (1/2000 Sigma-Aldrich). Epifluorescent imaging was performed using a high-resolution fluorescence imaging system (*Canberra Packard*). Confocal imaging of whole mount tissue was performed using a Zeiss LSM510 Meta Confocal microscope. 3D reconstructions were performed using Volocity from Improvision/Perkinelmer. Confocal microscopy acquisition and image analyses were performed at the CIAN core facility (Biology Dept., McGill University, Montréal).

Histology of adult eyes or pupal discs was processed following standard procedures. Histology of adult eyes was also prepared as modified from Gaengel and Mlodzik [50]. Fly heads were fixed and post fixed in a PB buffer. Incubation in the osmium solution was two hours. Incubation in 50% epon and 100% epon were performed overnight at 4°C. Semi-thin sections were cut using an ultratome and stained with 0.5% toluidine blue.

### Scanning Electron Microscopy

Flies were prepared following standard procedures for SEM. SEM was performed with critical point freezing in McGill Facility for Electronic Microscopy Research.

### Electroretinograms

ERGs were recorded by inserting a microelectrode into the eye, followed by dark adaptation for 5 min and stimulation for 2 sec with a strobe light.

### Determining Adult Retinal Length

Adult male flies were put into collars and fixed in Carnoy's solution (chloroform, ethanol and acetic acid) overnight at 4°C. Flies were then placed into methyl benzoate at 65°C, and then transferred to a mixed solution of methyl benzoate and paraffin wax (at 65°C). A final transfer was done to paraffin alone (again at 65°C). The flies-in-collars were placed into a cast with additional paraffin and allowed to cool. The wax is snapped off the collars ending up with heads embedded in wax all in the same orientation. Adult heads were cut using a microtome. Immunofluorescence was performed as previously mentioned. Tissue was stained with phalloidin and the length of retina was measured at the midpoint of the retina.

### Rhabdomere length analysis

Histological sections of fly heads were prepared from paraffin wax embedded material. The following protocol is modified from Shcherbata et al. 2007. Briefly, flies were placed in Heisenberg fly collars (Model #10731, 4M Instrument & Tool LLC, New York) and fixed in Carnoy's solution (6∶3∶1 ethanol (EtOH):chloroform:glacial acetic acid) overnight at 4°C; then further dehydrated in 100% EtOH (2×10′) at room temperature and, finally, infiltrated with paraffin (Poly/Fin, Triangle Biomedical Sciences, Inc) using the following procedure: Samples were placed in methyl benzoate for 30′ at 65°C, then transferred to a methyl benzoate:paraffin solution (1∶1) and further incubated at 65°C for 30′, and finally placed in paraffin alone at 65°C for an additional 30′. Afterwards samples were placed in casts then filled with melted paraffin (65°C). Once cooled and solidified, the collars were removed and frontal sections of the fly heads were cut with a rotary microtome (Leica 820 Histocut) at an 8 µm thickness. Paraffin was removed with xylene (2×4′) and the sections were rehydrated (100% EtOH 2×4′, 95% EtOH 1×3′, 70% EtOH 1×2′, H_2_O 1×1′) then stained with hematoxylin and eosin using standard protocols (H&E staining). Sections were covered with DPX Mountant (Fluka), cover-slipped and analyzed using confocal microscopy (Leica). Utilizing the fluorescence of the H&E stains, red channel images were viewed and analyzed. Sections corresponding to the midpoint of the fly head (from front to back) were used to obtain rhabdomere lengths. Rhabdomeres at the midpoint of the adult fly brain abutting the eye (the lamina) were measured using Leica software. Six to 14 fly heads were analyzed in a single sample preparation; and provided that both eyes were intact this corresponded to up to 12 to 28 measurements per preparation.

### Over-expression of *Dg* in wild type fly eyes

To observe the DG over-expression phenotypes, *elav*-Gal4 flies were crossed with UAS-*Dg* flies. Wild type flies and flies containing *elav*-Gal4/+ and UAS-*Dg*/+ were used as the controls. The genotype of *Dg* over-expression flies is *elav*-gal4/UAS-*Dg*. The plastic sections were made as described above and at least 10 eyes have been prepared for each *Dg* over-expression and control sample. To minimize the error in measurement resulting from samples at different location in the eye, sections were chosen from similar depths within the retina as judged by the size of the section. The perimeter and area of each ommatidium were measured from ommatidia localized in the center of each section. Measurements were done with Northern Eclipse software (Empix) and tabulated with Microsoft Excel and analyzed statistically using StatView (*Abacus Concepts, Inc*.).

## Supporting Information

Text S1 Supplemental Information.
(0.04 MB DOC)

Figure S1 Dm-DG expressed in heterologous cells is recognized by antibody IIH6 DGextra-mRFP was transfected in DG-null cells differentiated from mouse ES cells and stained with mab IIH6, an antibody that blocks function of vertebrate DG [3]. mRFP (A′) co-localizes with IIH6 (A) immunoreactivity in the non-permeablized cells on the plasma membrane (A′).
(1.29 MB TIF)

Figure S2 Distribution of DG in the developing fly eye. The eye disc of the 3^rd^ instar larvae is immunolabeled with mab 24B10, that recognizes chaoptin, a neuron-specific membrane protein (1) that is uniformly expressed in photoreceptor cell (R cell) and axons as well as in the tips of R cells in these apical, optical sections (A, D). An antiserum to *Drosophila* DG [4] labeled the apical R cells in wild type (B) but not in *Df(2R)Dg* ^248^ deficient R cells (E). C is the merge of A and B; F is the merge of D and E. In basal regions of the imaginal disc (G–L) where axons emerge, 24B10-positive staining (G) is surrounded by and overlaps with an area that also expresses DG (H, I). In *Df(2R)Dg* ^248^ deficient imaginal discs the bundling of axons in the basal region appears normal as revealed by 24B10 labeling (J) despite the absence of DG (K). By 40% of pupal development DG is found along with 24B10 in the rhabdomere at the centre of each ommatidium (arrowheads, M–O), at the basal aspect of each R cell (white arrows, M and O) and in non-R cells (red arrows, N, O), that are likely inter-ommatidial cells, in the distal disc. In the proximal disc DG is found on cells that surround axons (arrows, Q and R).
(4.13 MB TIF)

Figure S3 Altered localization of the zonula adherens marker β-catenin in *Df(2R)Dg* ^248^ ommatidia. Small patch mosaic (50% p.d.) ommatidia were examined immunohistochemically. Lack of GFP (green) identifies the mosaic patches. (A–C) wild type mosaic ommatidium localizes β-catenin, magenta, at photoreceptor R cell contact points spanning the length of the ommatidium, A, distal, B, midpoint and C proximal. (D–E) A *Df(2R)Dg* ^248^ mosaic ommatidium shows a diffuse pattern of β-catenin, magenta, indicating disrupted polarity. D, distal, E, midpoint, F, proximal. Scale bar represents 6 µm.
(2.61 MB TIF)
