# Supplementary material for: Dystroglycan and Mitochondrial Ribosomal Protein L34 Regulate Differentiation in the Drosophila Eye
Source: PLoS One. 2010 May 5;5(5):e10488. doi: 10.1371/journal.pone.0010488 (PMC2864756; doi:10.1371/journal.pone.0010488)
Supplement: Text S1 — Supplemental Information. (0.04 MB DOC) [file pone.0010488.s001.doc]

# Supplemental Information

Yougen Zhan*^ab^, Nadia Y. Melian*^a^, Mario Pantoja^c^, Nicola Haines^a^, Hannele Ruohola-Baker^c^, **Charles W** Bourque^a^, Yong Rao^a^ and Salvatore Carbonetto^a,1^

*These authors contributed equally to this work

**SI Materials and Methods**

**Generation of DG_extra_-mRFP construct**

cDNA of DGextra encoding extracellular and transmembrane domains of *Drosophila* DG was PCR-amplified using sense 5’-GAG TAA GCT TAT GAG ATT CCA GTG GTT CTT ATC GGC-3’ and antisense primer 5’-GAG TAG ATC TAG CCA GTG CAG GCA ACA GGC AAT G-3’ from genomic DNAs of UAS-DG flies, and inserted in frame into HindIII and BglII sites 5’ upstream of mRFP in pcDNA3 vector (provided by Dr. Keith Murai, McGill University). The sequence was confirmed by The McGill University and Genome Québec Innovation Centre

**DG_extra_-mRFP transfection and Immunocytochemistry**

DG-null 3C12 embryonic stem cells engineered previously [1] were differentiated in culture as described [2] and were transfected with DGextra-mRFP and mRFP under control of CMV promoter in pcDNA3 vector using Fugene reagent (*Roche*) according to manufacturer’s recommendations. A mouse antibody IIH6, which recognizes the functional carbohydrates of α-DG in vertebrates, was used to visualize the glycosylation of the transfected *Drosophila* DG containing the extracellular and transmembrane sequences fused to mRFP (DG_extra_-mRFP). Procedures for immunocytochemistry were similar to the described previously [2]. Briefly transfected cells were fixed in 4% paraformaldehyde, washed and incubated with IIH6 without permeablization and then incubated with fluorescein-labeled secondary antibody. Cells were observed with a Zeiss Axioskop epifluorescent microscope and images were captured with a Retiga 1300 monochrome 10 bit digital camera (*Qimaging Corp.*) and analyzed with Northern Eclipse software (*Empix*).

## SI References

1. Côté PD, Moukhles H, Lindenbaum M, Carbonetto S (1999) Chimaeric mice deficient in dystroglycans develop muscular dystrophy and have disrupted myoneural synapses. Nat Genet 23: 338-342.

2. Zhan Y, Tremblay MR, Melian N, Carbonetto S (2005) Evidence that dystroglycan is associated with dynamin and regulates endocytosis. J Biol Chem 280: 18015-18024.

3. Gee SH, Montanaro F, Lindenbaum MH, Carbonetto S (1994) Dystroglycan-alpha, a dystrophin-associated glycoprotein, is a functional agrin receptor. Cell 77: 675-686.

4. Deng WM, Schneider M, Frock R, Castillejo-Lopez C, Gaman EA, et al. (2003) Dystroglycan is required for polarizing the epithelial cells and the oocyte in Drosophila. Development 130: 173-184.

**SI Figure Legends**

***Figure S1. Dm-DG expressed in heterologous cells is recognized by antibody IIH6***

DG_extra_-mRFP was transfected in DG-null cells differentiated from mouse ES cells and stained with mab IIH6, an antibody that blocks function of vertebrate DG [3]. mRFP (A’) co-localizes with IIH6 (A) immunoreactivity in the non-permeablized cells on the plasma membrane (A’’).

# Figure S2. Distribution of DG in the developing fly eye.

The eye disc of the 3rd instar larvae is immunolabeled with mab 24B10, that recognizes chaoptin, a neuron-specific membrane protein (1) that is uniformly expressed in photoreceptor cell (R cell) and axons as well as in the tips of R cells in these apical, optical sections (A**,** D). An antiserum to *Drosophila* DG [4] labeled the apical R cells in wild type (B) but not in *Df(2R)Dg^248^* deficient R cells (E). C is the merge of A and B; F is the merge of D and E. In basal regions of the imaginal disc (G-L) where axons emerge, 24B10-positive staining (G) is surrounded by and overlaps with an area that also expresses DG (H, I). In *Df(2R)Dg^248^* deficient imaginal discs the bundling of axons in the basal region appears normal as revealed by 24B10 labeling (J) despite the absence of DG (K). By 40% of pupal development DG is found along with 24B10 in the rhabdomere at the centre of each ommatidium (arrowheads, M-O), at the basal aspect of each R cell (white arrows, M and O) and in non-R cells (red arrows, N, O), that are likely inter-ommatidial cells, in the distal disc. In the proximal disc DG is found on cells that surround axons (arrows, Q and R).

***Figure S3. Altered localization of the zonula adherens marker β-catenin in Df(2R)Dg^248^ ommatidia.*** Small patch mosaic (50% p.d.) ommatidia were examined immunohistochemically. Lack of GFP (green) identifies the mosaic patches. (A-C) wild type mosaic ommatidium localizes β-catenin, magenta, at photoreceptor R cell contact points spanning the length of the ommatidium, A, distal, B, midpoint and C proximal. (D-E) A *Df(2R)Dg^248^* mosaic ommatidium shows a diffuse pattern of β-catenin, magenta, indicating disrupted polarity. D, distal, E, midpoint, F, proximal. Scale bar represents 6μm.
