# Supplementary figures and images for: Dystroglycan and Mitochondrial Ribosomal Protein L34 Regulate Differentiation in the Drosophila Eye
Source: PLoS One. 2010 May 5;5(5):e10488. doi: 10.1371/journal.pone.0010488 (PMC2864756; doi:10.1371/journal.pone.0010488)

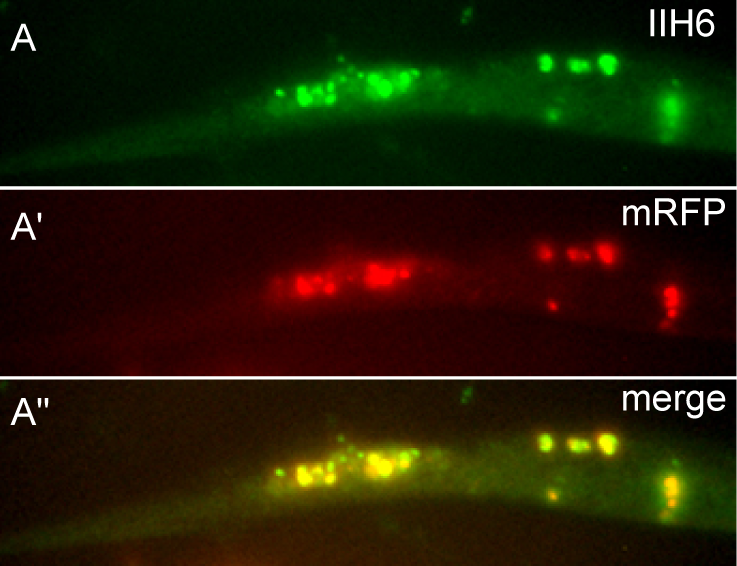

Supplement: Figure S1 — Dm-DG expressed in heterologous cells is recognized by antibody IIH6 DGextra-mRFP was transfected in DG-null cells differentiated from mouse ES cells and stained with mab IIH6, an antibody that blocks function of vertebrate DG [3]. mRFP (A′) co-localizes with IIH6 (A) immunoreactivity in the non-permeablized cells on the plasma membrane (A′). (1.29 MB TIF) [file pone.0010488.s002.tif]

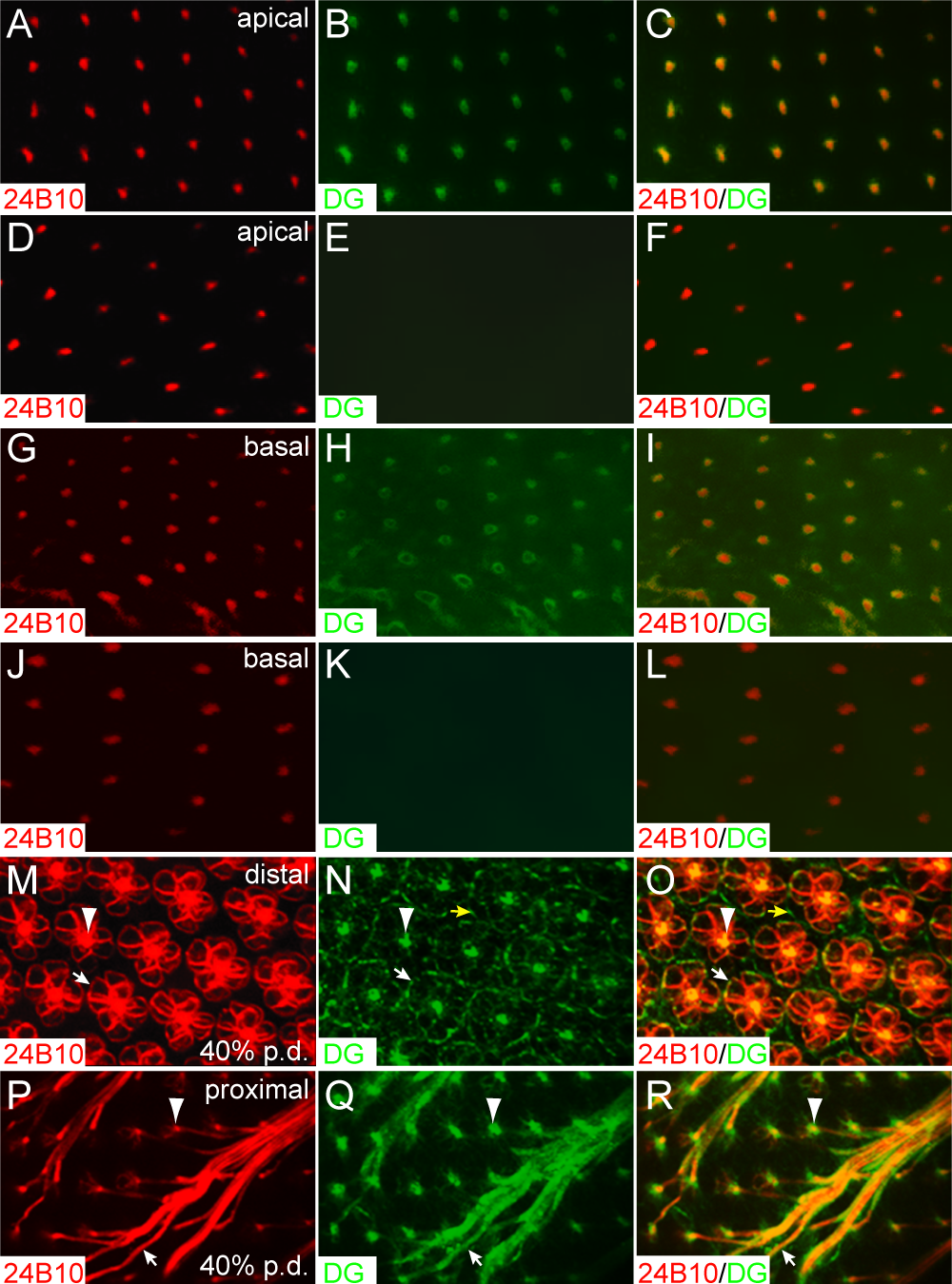

Supplement: Figure S2 — Distribution of DG in the developing fly eye. The eye disc of the 3rd instar larvae is immunolabeled with mab 24B10, that recognizes chaoptin, a neuron-specific membrane protein (1) that is uniformly expressed in photoreceptor cell (R cell) and axons as well as in the tips of R cells in these apical, optical sections (A, D). An antiserum to Drosophila DG [4] labeled the apical R cells in wild type (B) but not in Df(2R)Dg 248 deficient R cells (E). C is the merge of A and B; F is the merge of D and E. In basal regions of the imaginal disc (G–L) where axons emerge, 24B10-positive staining (G) is surrounded by and overlaps with an area that also expresses DG (H, I). In Df(2R)Dg 248 deficient imaginal discs the bundling of axons in the basal region appears normal as revealed by 24B10 labeling (J) despite the absence of DG (K). By 40% of pupal development DG is found along with 24B10 in the rhabdomere at the centre of each ommatidium (arrowheads, M–O), at the basal aspect of each R cell (white arrows, M and O) and in non-R cells (red arrows, N, O), that are likely inter-ommatidial cells, in the distal disc. In the proximal disc DG is found on cells that surround axons (arrows, Q and R). (4.13 MB TIF) [file pone.0010488.s003.tif]

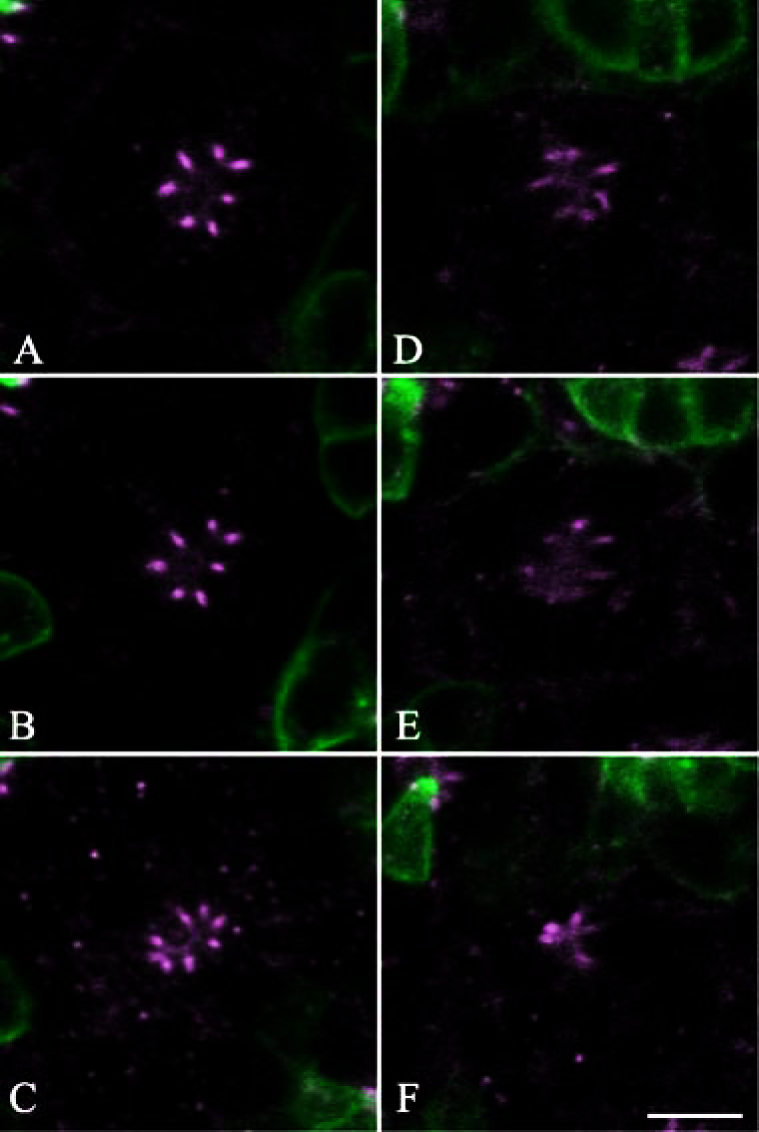

Supplement: Figure S3 — Altered localization of the zonula adherens marker β-catenin in Df(2R)Dg 248 ommatidia. Small patch mosaic (50% p.d.) ommatidia were examined immunohistochemically. Lack of GFP (green) identifies the mosaic patches. (A–C) wild type mosaic ommatidium localizes β-catenin, magenta, at photoreceptor R cell contact points spanning the length of the ommatidium, A, distal, B, midpoint and C proximal. (D–E) A Df(2R)Dg 248 mosaic ommatidium shows a diffuse pattern of β-catenin, magenta, indicating disrupted polarity. D, distal, E, midpoint, F, proximal. Scale bar represents 6 µm. (2.61 MB TIF) [file pone.0010488.s004.tif]
